# Preparation of hydroxyapatite nanofibers by using ionic liquids as template and application in enhancing hydrogel performance

**DOI:** 10.3389/fbioe.2023.1247448

**Published:** 2023-08-02

**Authors:** Xiuli Ren, Zepeng Liang, Xingjun Zhao

**Affiliations:** College of Pharmacy, Jinzhou Medical University, Jinzhou, China

**Keywords:** hydroxyapatite, biomaterials, nanofibers, hydrogel, ionic liquids

## Abstract

**Introduction:** Hydroxyapatite (HAP or HA) nanofibers are very attractive in the field of biomedical engineering. However, templates used for preparing HAP nanofibers are usually hydrophobic molecules, like fatty acids and/or surfactants, which are difficult to remove and potentially toxic. Therefore, it is important to develop a green approach to prepare HAP nanofibers.

**Methods:** Imidazolium-based ionic liquids (ILs) were used as templates to control the crystallization of HAP. The obtained HAP nanofibers were composited into polyvinyl alcohol-sodium alginate (PVA-Alg) hydrogel (HAP@H). The rheological performance, stretching, and compression properties were tested. Scanning electron microscope (SEM), high resolution transmission electron microscope (HRTEM), X-ray diffraction (XRD), Fourier-transform infrared (FT-IR), and differential scanning calorimetry (DSC) were adopted to characterize the morphology, size, crystallographic orientations, and phase of HAP@H.

**Results:** HAP nanofibers with a length of ∼50 μm were harvested. The DSC results proved that water loss temperature increased from 98°C (for pure hydrogel) to 107°C (for HAP@H). Also, HAP@H hydrogel presented much better porous structure, tensile performance, and compressive performance than that of pure hydrogel.

**Discussion:** The morphology, size, and growth direction of HAP could be modulated easily by altering the alkyl chain length of ILs’ cations. This is possibly due to face-specific adsorption of imidazolium moieties on HAP nanocrystals. The enhancing performance of HAP@H is probably due to the composited highly oriented HAP nanofibers.

## 1 Introduction

Because of the highly anisotropic geometry and size confinement, nanofibers have attracted much attention in various fields, such as catalysis, sensors, solar cells, lithium batteries, novel probe microscopy tips, functional nanostructured materials, and super-strong and tough composites (Zhang et al., 2014; Levitt et al., 2019; Stojanov and Berlec, 2020; Du et al., 2023). Among various kinds of nanofibers, hydroxyapatite (HAP) nanofibers are attractive in drug or gene delivery (Doan et al., 2013; Tsai et al., 2018), bone repair (Ao et al., 2017; Li et al., 2018), tissue engineering (Chen et al., 2019; Zheng et al., 2021), and functional composite materials (Cao et al., 2020; Dong and Zhu, 2021). The methods of preparing HAP nanofibers include homogeneous precipitation and hydrothermal methods (Zhang and Darvell, 2010; Costa et al., 2012; Zhuang et al., 2013; Wang et al., 2020). However, up to now, the reported method of preparing HAP nanofibers usually used templates involving hydrophobic molecules, like fatty acids and/or surfactants, which are difficult to remove and may even be toxic. Therefore, it is important to develop a green approach to fabricate HAP nanofibers.

Ionic liquids (ILs) are very attractive because of their outstanding advantages like negligible vapor pressure, thermal stability, chemical design, and recyclability (Gomes et al., 2019; Singh and Savoy, 2020; Kaur et al., 2022). ILs have been considered as green, environmentally benign solvents (Zhao et al., 2009; Curreri et al., 2021). The chemical stability and structure of ILs can be designed. This virtue is potentially useful in improving the physicochemical properties of nanoparticles and/or in constructing or templating nanomaterials (Zhao et al., 2009; Hayes et al., 2015; He and Alexandridis, 2015; Curreri et al., 2021).

**FIGURE 1:**
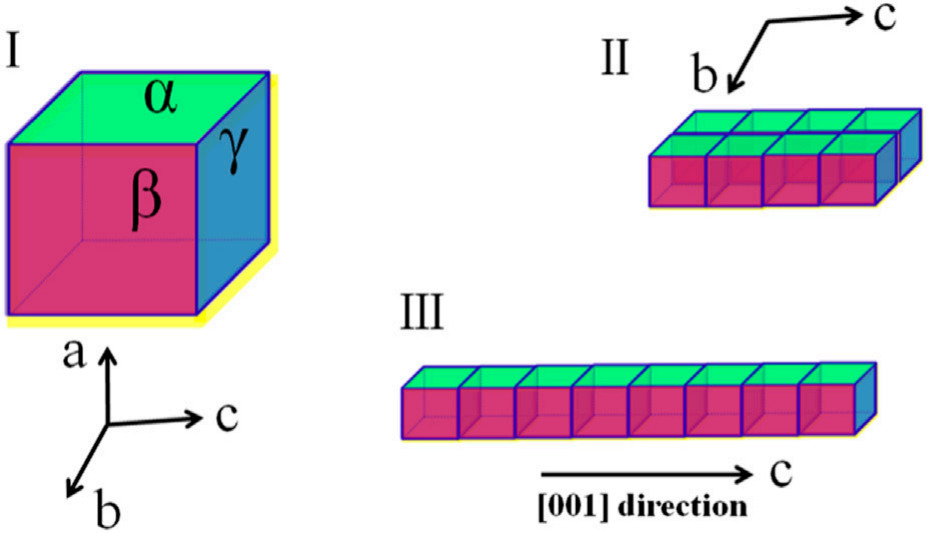
Three schematic growth routes of HAP crystal: **(I)** without a growth modifier; **(II)** with modifiers which selectively adsorbed on α facet; and **(III)** with modifiers that selectively adsorbed on both α and β facets.

Herein, in this work, we proposed a strategy of using ILs as green templates to facilitate the controlled crystallization of HAP. As illustrated in Figure 1I, without a crystal growth modifier, the HAP clusters would grow in the three orientations along the *a*-, *b*-, and *c*-axis, respectively. However, as all known, HAP crystals are usually oriented in the *a*-axis (Figure 1II) in dental enamel, while they are *c*-axis-oriented (Figure 1III) in bone. Highly *c*-axis-oriented HAP crystals have been reported to have higher biomechanical properties than other oriented HAP. After collecting *c*-axis-oriented HAP nanofibers, the porous structure, tensile performance, and compressive performance of nanofibers composited HAP@H were also characterized and tested.

## 2 Experiment

### 2.1 Materials

**FIGURE 2:**
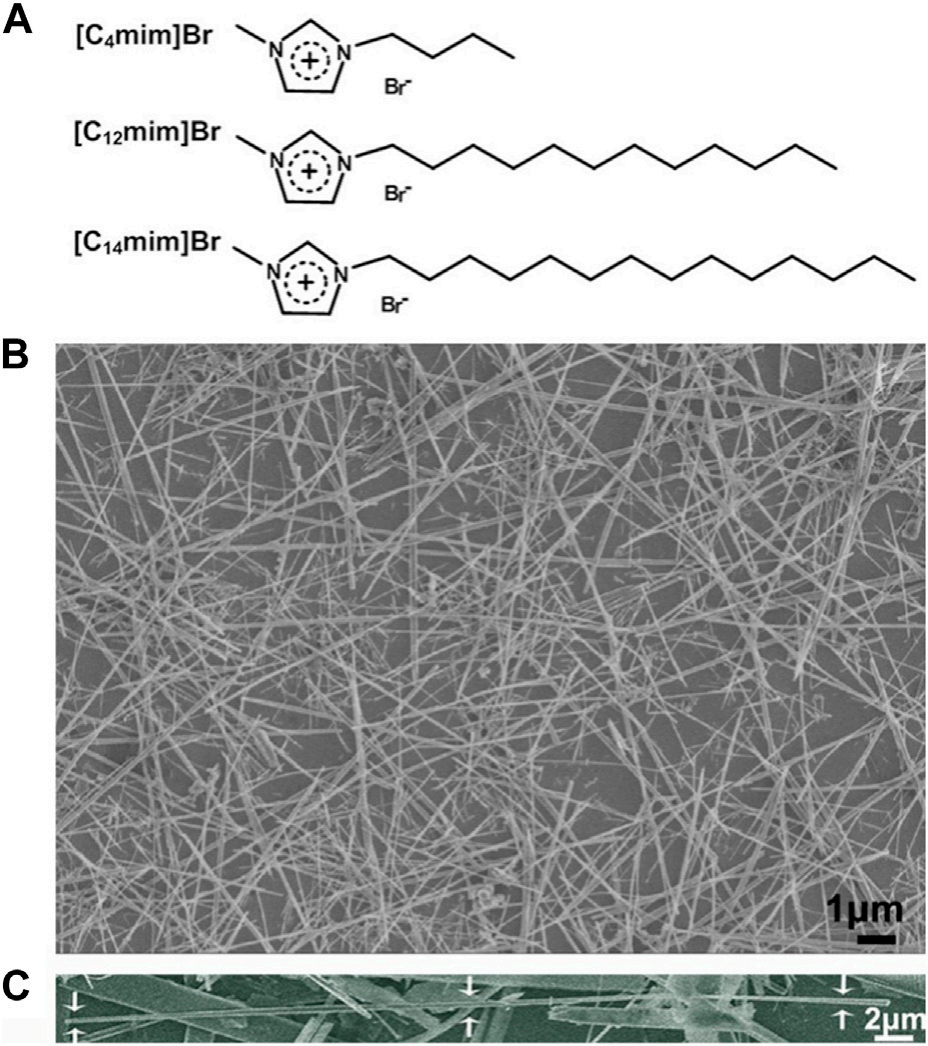
**(A)** Schematic structure of three ILs used in this work; **(B)** SEM image of HA fibers templated by [C_14_mim]Br (ILHA-3); and **(C)** SEM image of one magnified ILHA-3 fiber with a length of ∼45 μm.

Calcium chloride (ACS reagent, ≥96.0%), sodium dihydrogen phosphate (ACS reagent, ≥98.0%), sodium hydroxide (ACS reagent, ≥97.0%), polyvinyl alcohol (PVA, Mw 9,000–10,000), and sodium alginate (Alg) were all purchased from Sigma-Aldrich, St. Louis, MO, United States. Deionized water (18.3 MΩ cm) was used to prepare all aqueous solutions. All ILs 1-butyl-3-methylimidazolium bromide [C_4_mim]Br; 1-dodecyl-3-methylimidazolium bromide [C_12_mim]Br; and 1-tetradecyl-3-methylimidazolium bromide [C_14_mim]Br were prepared and purified using the similar procedure described previously by Zhao et al. (2009), and their structure was illustrated in Figure 2A.

### 2.2 Mineralization of HAP crystals

The mineralization of HAP crystals was carried out using the hydrothermal precipitation method similar to the literature (Hao et al., 2013). In all experiments, the initial Ca^2+^ and PO_4_^3-^ concentrations were fixed at 0.1 M and 0.06 M, respectively. CaCl_2_ and NaH_2_PO_4_•·2H_2_O were used as Ca^2+^ and PO_4_^3-^ reservoirs, respectively. A series of CaCl_2_/NaH_2_PO_4_.2H_2_O/ILs solution was prepared, and the concentration of ILs was fixed at 10 mg mL^-1^. In a typical procedure, 1.0 g [C_14_mim]Br and 0.48 g NaOH were dissolved in 50 mL water. Subsequently, a solution of CaCl_2_ (25 mL, 0.1 M) and the other solution of NaH_2_PO_4_.2H_2_O (25 mL, 0.06 M) were added. The obtained mixtures were stirred using vortex mixing (IKA, Vortex, Genius 3) for 10 min. Then, the resultant mixtures were transferred to a 200 mL teflon-lined stainless autoclave container at 200°C for 7 h. After that, the container cooled naturally to room temperature prior to harvest the crystals by centrifugation. Finally, the collected crystals were rinsed several times with deionized water and air-dried for further analyses. All experiments were repeated at least twice. A control experiment was also carried out in the absence of ILs. The feed composition and designations of the obtained apatite crystals in the absence or presence of ILs are listed in Table 1.

**TABLE 1:** Group designation of the synthetic HA in the absence and presence of ILs.

Sample	ILs/1.0 g	CaCl_2_/g	NaH_2_PO_4_·2H_2_O/g	NaOH/g	H_2_O/mL	Morphology
HA-0	0	1.11	0.936	0.48	100	Needles
ILHA-1	[C_4_mim]Br	1.11	0.936	0.48	100	Flakes
ILHA-2	[C_12_mim]Br	1.11	0.936	0.48	100	Conjugate fibers
ILHA-3	[C_14_mim]Br	1.11	0.936	0.48	100	Fibers

### 2.3 Characterization

The morphologies of the products were investigated by scanning electron microscopy (SEM, Hitachi, S4800, Tokyo, Japan). To identify the composition of the synthetic products, Fourier-transform infrared spectroscopy (FTIR) was performed by using a SHIMADZU spectrum system (SHIMADZU, Kyoto, Japan) with a resolution of 4.00 cm^−1^. X-ray diffraction (XRD) patterns were collected on a SHIMADZU X-lab 6000 X-ray powder diffractometer with Cu Kα radiation to confirm the phase and structure of the formed crystals. High-resolution transmission electron microscopy (HRTEM, FEI, Tecnai G^2^ F20 STwin, USA) was adopted to investigate the microstructure together with the Fourier-transform (FFT) method. The differential scanning calorimetry (DSC) curves were performed on TGA/DSC1/1,100 (Mettler Toledo, Switzerland).

### 2.4 Preparation of HAP@H

To prepare the hydrogel, 0.53 g PVA was dissolved in 10 mL of distilled water and placed in an oven (80°C) for 1 h according to the previous work (Zhang et al., 2020; Zhang et al., 2021; Ren et al., 2023). Then, 0.1 g Alg was added into the PVA solution and the mixture was stirred for 1 h under constant temperature of 70°C to form a homogenous solution. HAP@H was synthesized by adding 0.05 g lyophilized HAP powder into the obtained solution under stirring.

### 2.5 Hydrogel performance testing

Rheological performances of different groups, including PVA solution, PVA-Alg hydrogel, and HAP@H, were carried out by investigating the changes in flowability of tilted small bottles according to the reported work (Chen et al., 2020; Ding et al., 2021). Macroscopic morphologies of pure hydrogel and HAP@H were observed. In order to investigate the porous structure of the obtained hydrogel, the freeze-dried gel was sliced and observed by micrography. The tensile and elastic properties of HAP@H were also tested by stretching and knotting treatments. Compression and swelling properties were performed on the hydrogel and the lyophilized hydrogel.

## 3 Results and discussion

### 3.1 Crystallization and characterization of HAP

The structures of the ILs ([C_4_mim]Br, [C_12_mim]Br, and [C_14_mim]Br) used in controlling HAP crystallization are illustrated in Figure 2A. The alkyl chain length of imidazolium cations ranged from C4 to C14. A control experiment was also carried out in the absence of ILs. The feed composition and designations of the groups are listed in Table 1.

The SEM image of HA crystals templated by [C_14_mim]Br (ILHA-3) is shown in Figure 2B, and it is clear that these ILHA-3 were nanofibers with lengths ranging from 20 to 50 μm. A single ILHA-3 fiber is shown in Figure 2C, as indicated by the arrows. This fiber had a length of about 45 μm and a diameter of about 0.3 μm. These results demonstrated that [C_14_mim]Br could successfully template HAP into highly *c*-axis-oriented nanofibers with length of tens of microns.

**FIGURE 3:**
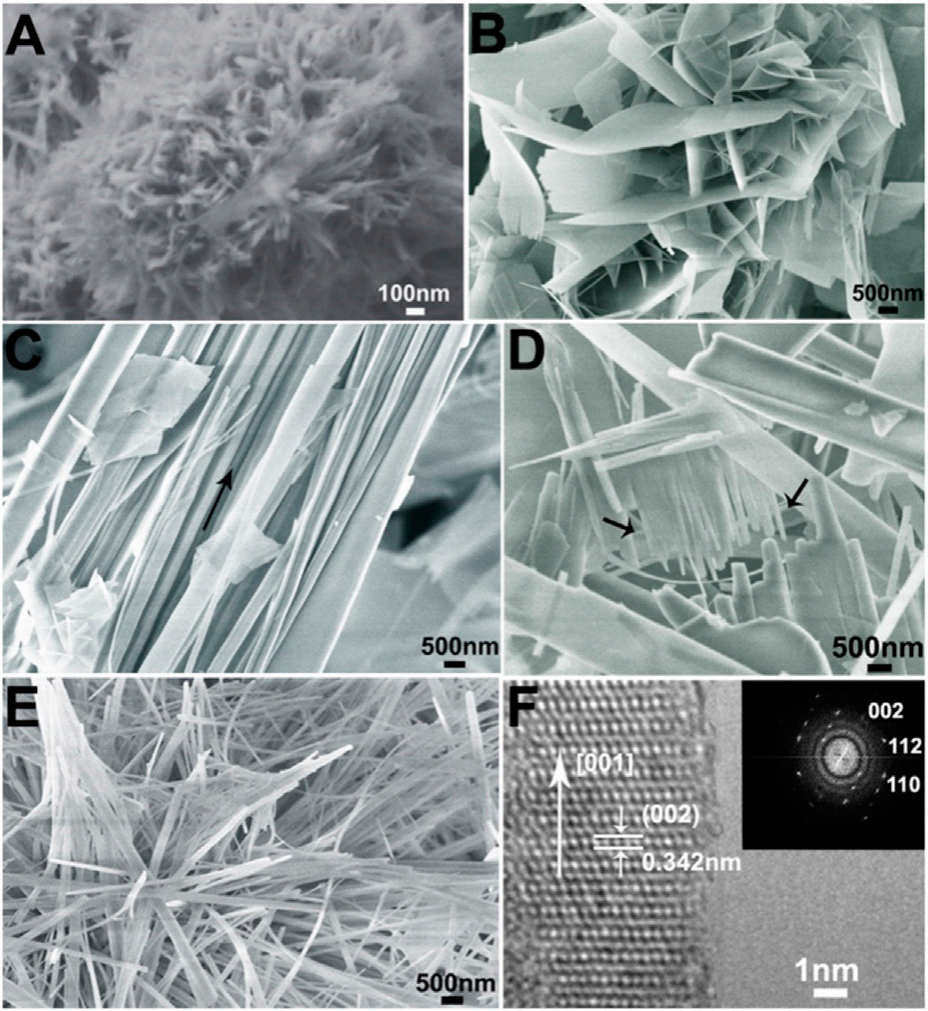
SEM images of samples: **(A)** HA0 (blank control); **(B)** ILHA-1 (templated by [C_4_mim]Br); **(C)** and **(D)** ILHA-2 (templated by [C_12_mim]Br); and **(E)** ILHA-3. **(F)** HRTEM and the corresponding Fourier-transform diffractogram (inset) of the ILHA-3 nanofiber.

In order to investigate the effect of the alkyl chain length of ILs cations on the mineralization of HAP, the morphology of the samples from the groups (listed in Table 1) of HA-0, ILHA-1, ILHA-2, and ILHA-3 was characterized by SEM and TEM (Figure 3). In the blank control group (HA-0), the products were aggregates of nanoneedle (about 300 nm in length and 40 nm in width). This result suggests that it is difficult to get high-aspect ratio HAP crystals in the absence of the crystallization template. Also, in the group of ILHA-1, flake-shaped HA crystals were obtained. This result suggests that [C_4_mim]Br could inhibit the growth orientation of the *a*-axis; thus, the HAP would grow along the *b*-axis and *c*-axis orientation to form thin flake-shaped crystals. Figure 3C indicates that the HAP crystals obtained in the ILHA-2 group were mainly conjugated fibers. It could be inferred that these conjugated fibers might be generated by incomplete flaky crystal cracking. Also, this was proved by Figure 3D, indicated by the arrows, that the front end of the flaky crystal cracked into fibrous crystals. Moreover, in the ILHA-3 group, the SEM image in Figure 3E proved that the obtained HAP crystals were nanofibers. The HRTEM image in Figure 3F further suggests that the <001> orientation (*c*-axis) and 0.342 nm crystal lattice are ascribed to (002) crystal plane. These results were further supported by Fourier-transform diffractogram inserted in Figure 3F.

**FIGURE 4:**
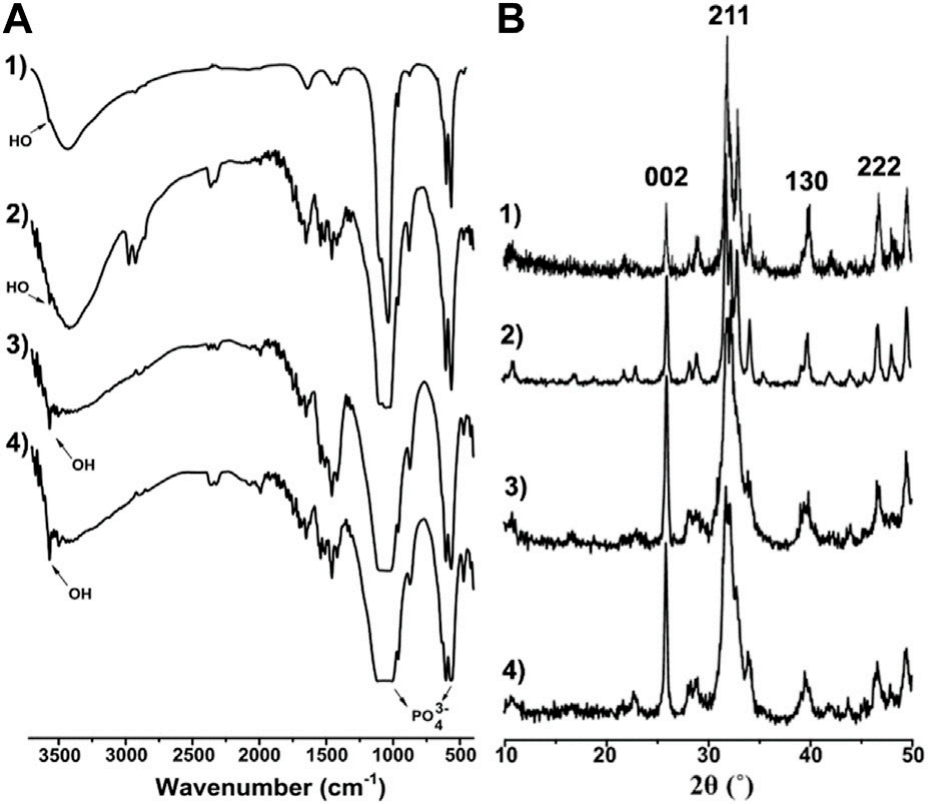
IR spectra **(A)** and XRD patterns **(B)** of 1) HA-0; 2) ILHA-1; 3) ILHA-2; and 4) ILHA-3.

Figure 4 provides the IR and XRD characterization of the samples. The IR spectra in Figure 4A indicate that the absorption peaks at ∼3,500 cm^-1^, which are ascribed to the OH of HAP crystals, had an obvious increase in intensity from group HA-0 to ILHA-3. The increased intensity of OH absorption usually indicates an increase in crystallinity or orientation of HAP. This inference was further proved by the XRD patterns shown in Figure 4B. It is clear that the intensity of the (002) crystal plane had been obviously increased, which signifies the increased orientation of HAP along the *c*-axis (<001>).

**FIGURE 5:**
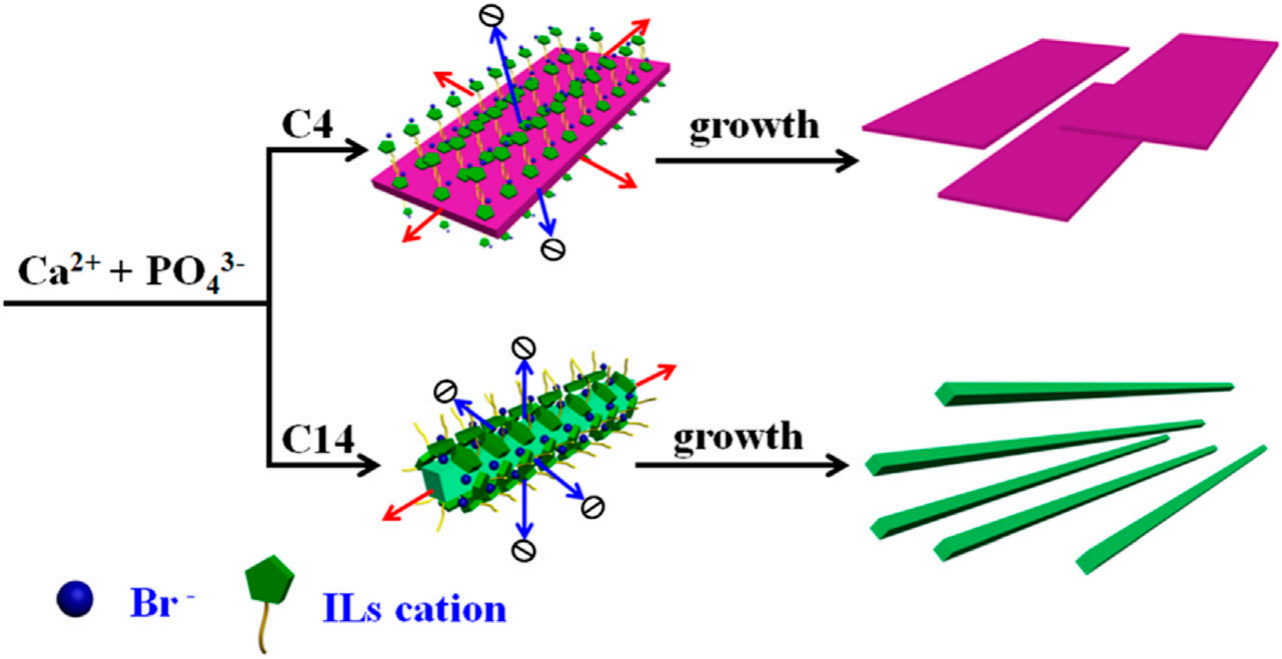
Schematic crystal growth process of hydroxyapatite nanoflakes and nanofibers under the control of [C_4_mim]Br and [C_14_mim]Br, respectively.

In order to further discuss the role of the ILs template in controlling the crystallization of HAP, the schematic crystal growth processes of HAP nanoflakes templated by [C_4_mim]Br and nanofibers templated by [C_14_mim]Br are shown in Figure 5. It is suggested that the [C_4_mim]Br might have adhered to the surface of the crystals; therefore, it could inhibit the growth along the *a*-axis. Thus, the ILHA-1 crystals, controlled by [C_4_mim]Br, grew along the *b*-axis and *c*-axis to form nanoflakes. However, for using [C_14_mim]Br as a template, these ILs would inhibit the growth both along the *a*-axis and *b*-axis; thus, the ILHA-3 group was controlled to grow along the *c*-axis to form nanofibers.

### 3.2 Preparation of HAP@H hydrogel and their performance testing

**FIGURE 6:**
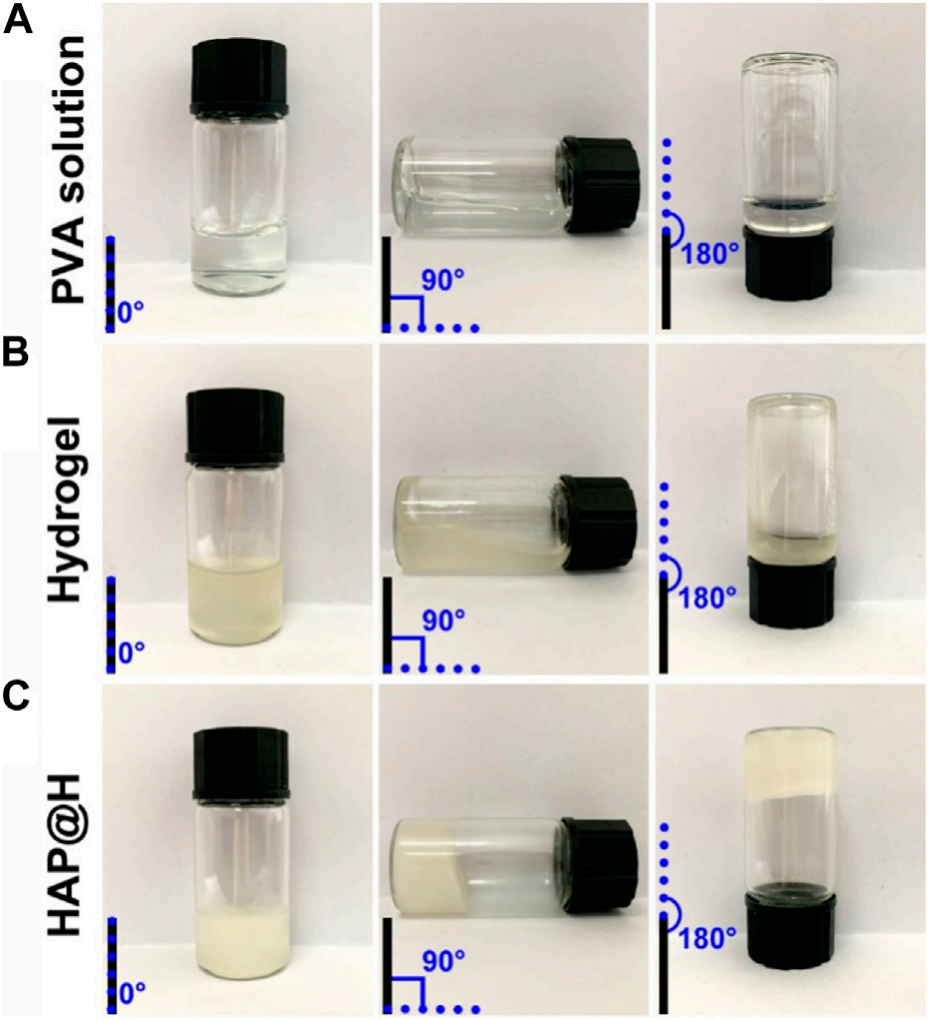
Rheological performance photos of different groups: **(A)** PVA solution, **(B)** PVA-Alg hydrogel, and **(C)** HAP@H.

Rheological performance testing of PVA solution, PVA-Alg hydrogel, and HAP@H was carried out by testing flowability changes in tilted small bottles, as shown in Figure 6. It is clear that the pure PVA solution had good flowability (Figure 6A). Although the freshly prepared pure hydrogel also presented flowability (Figure 6B), the gel hanging on the bottom and wall of the bottle indicates that the viscosity of gel was much higher than that of pure PVA. It should be noted that pure hydrogel was still not solidified and needed aging time. However, the newly prepared HAP@H presented rapid gel curing performance and did not require a long gel aging time (Figure 6C).

**FIGURE 7:**
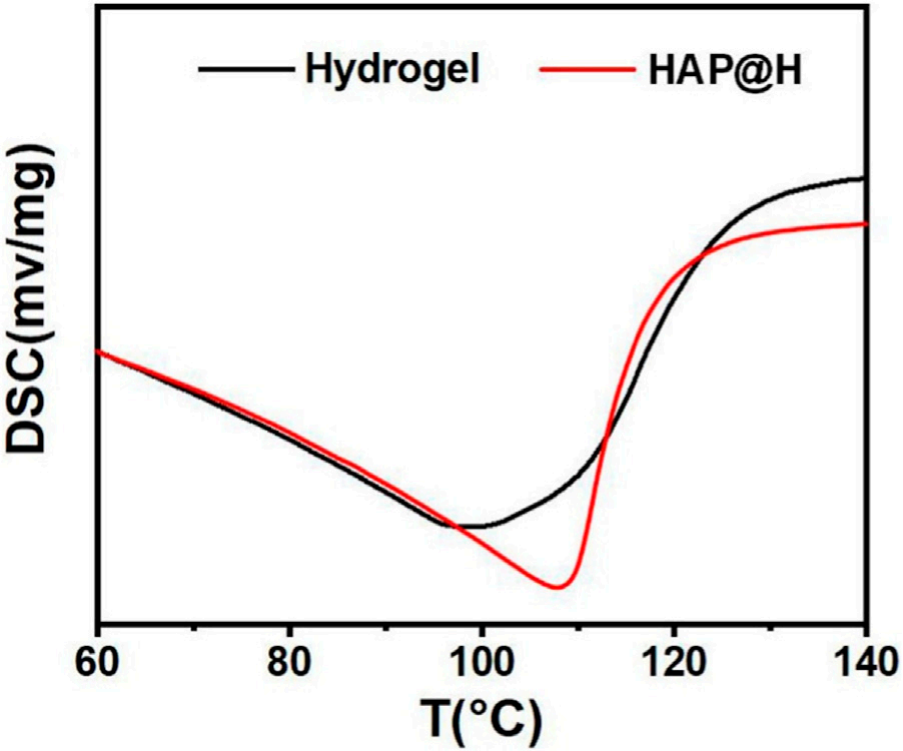
DSC curves of blank hydrogel and HAP-composited hydrogel.

In order to investigate the mechanism behind the rheological performance difference between pure hydrogel and HAP@H, the DSC test was performed on the samples. The DSC curves (Figure 7) proved that HAP@H had an increase (from 98°C to 107°C) in the water loss temperature compared to pure hydrogel. This might suggest that the strong interaction of hydrogen bonds between the HAP crystals and hydrogel network molecules enhanced the moisture retention ability.

**FIGURE 8:**
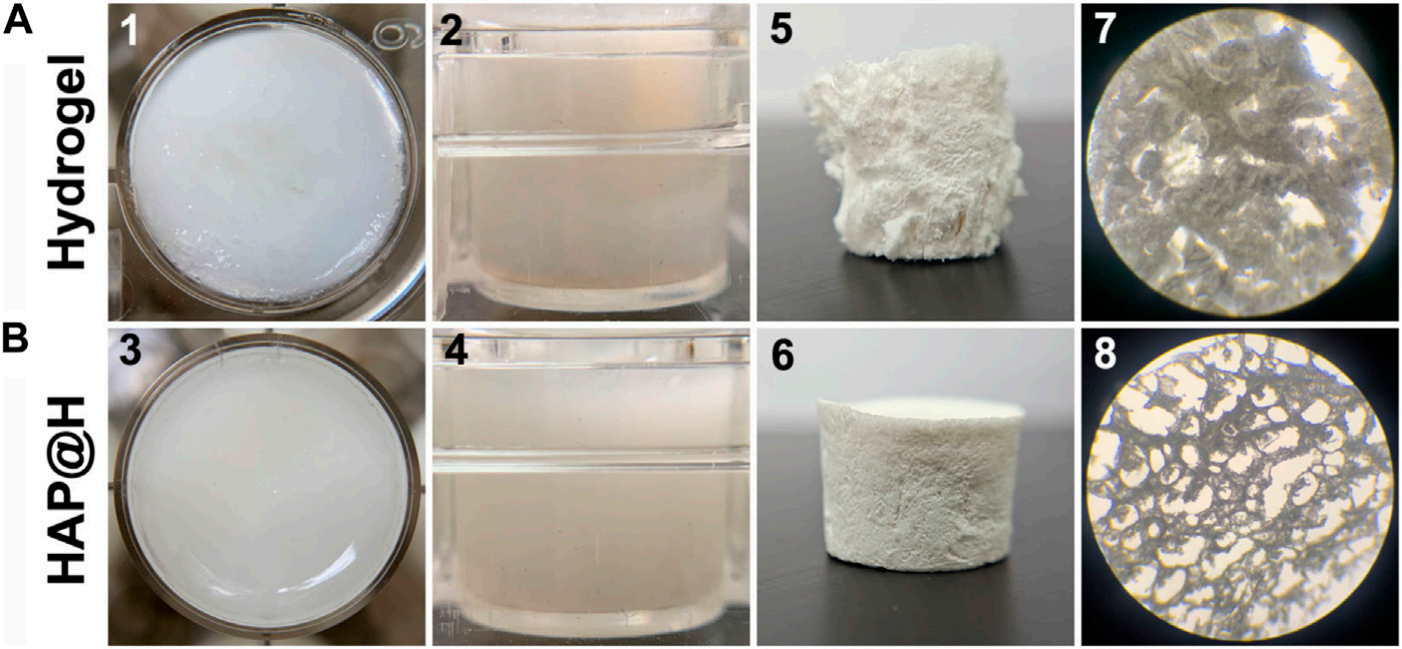
Macroscopic photos (1–6) and micrography images (7, 8) of pure hydrogel **(A)** and HAP@H **(B)**; 1–4 are the photos of freshly prepared hydrogels, and 5–8 are the images for the freeze-dried hydrogels; 1 and 3: top view; 2 and 4: side view.

After gel aging, pure hydrogel and HAP@H, placed in 24-well plates, presented several differences, as shown in Figure 8. First, HAP@H presented a more even and compact hydrogel appearance than that of pure hydrogel. Unlike the compact and uniform appearance for HAP@H shown in Figure 8B(3) and Figure 8B(4), the photos for pure hydrogel showed a less compact (Figure 8A(1)) and not heterogeneous appearance (Figure 8A(2)). Also, these appearance differences were further proved by the lyophilized gel’s morphology comparison. Figure 8A(5,7) shows that the lyophilized pure hydrogel did not have a uniform porous structure, while lyophilized HAP@H presented a good and uniform porous structure, as shown in Figure 8B(6, 8).

**FIGURE 9:**
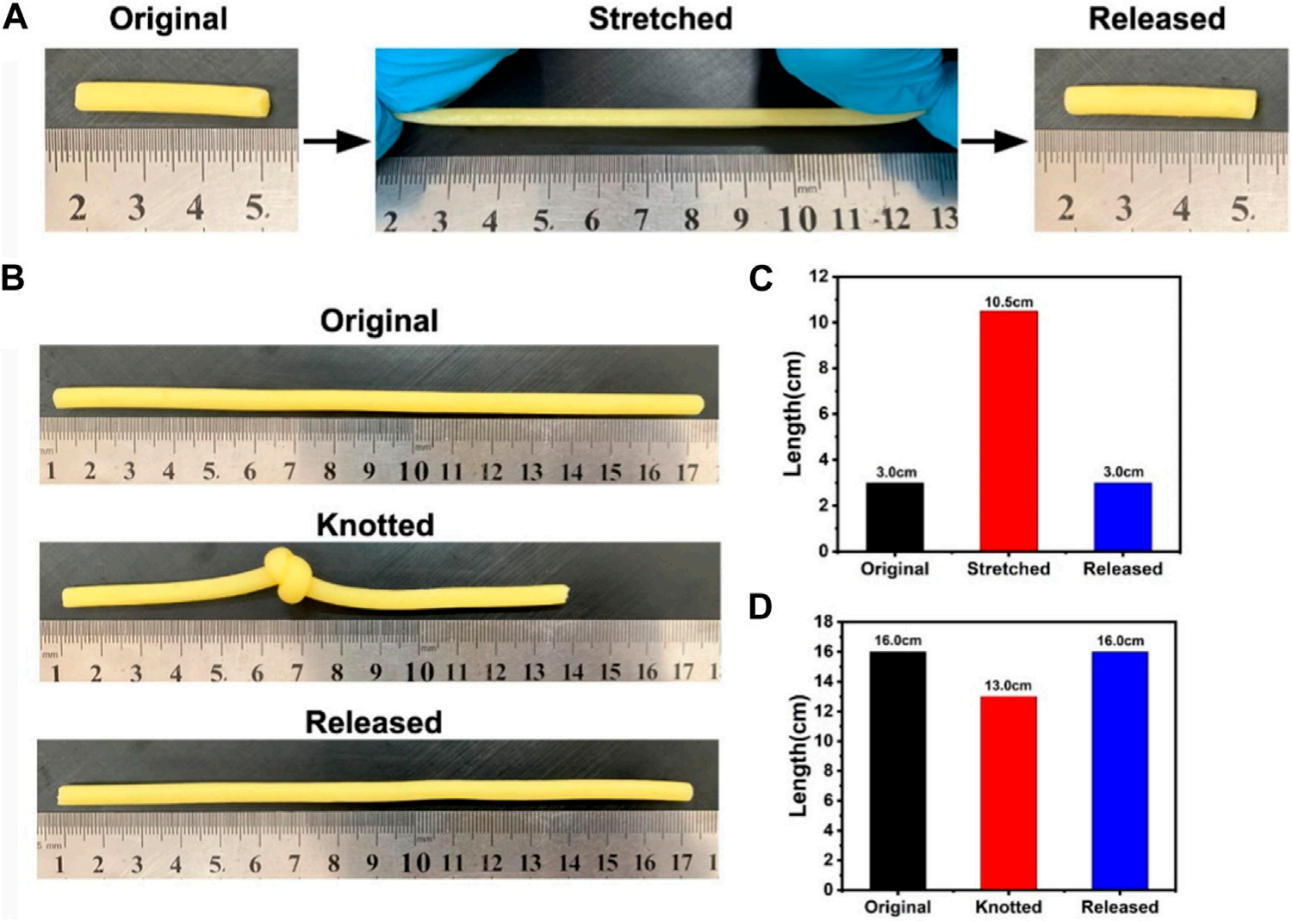
**(A, B)** Photographs of hydrogel stretching and knotting tests; **(C, D)** Lengths of hydrogels before and after stretching and knotting treatments, respectively.

The tensile and elastic properties of HAP@H were also tested by stretching and knotting treatments, as indicated in Figure 9. The HAP@H in 3.0 cm length could be starched to the length of 10.5 cm. After releasing, the sample restored to the original 3.0 cm length (Figures 9A,C). Also, the knotting treatment for HAP@H (shown in Figures 9B,D) revealed that the knot–untie operation did not change the shape and length of the gel sample.

**FIGURE 10:**
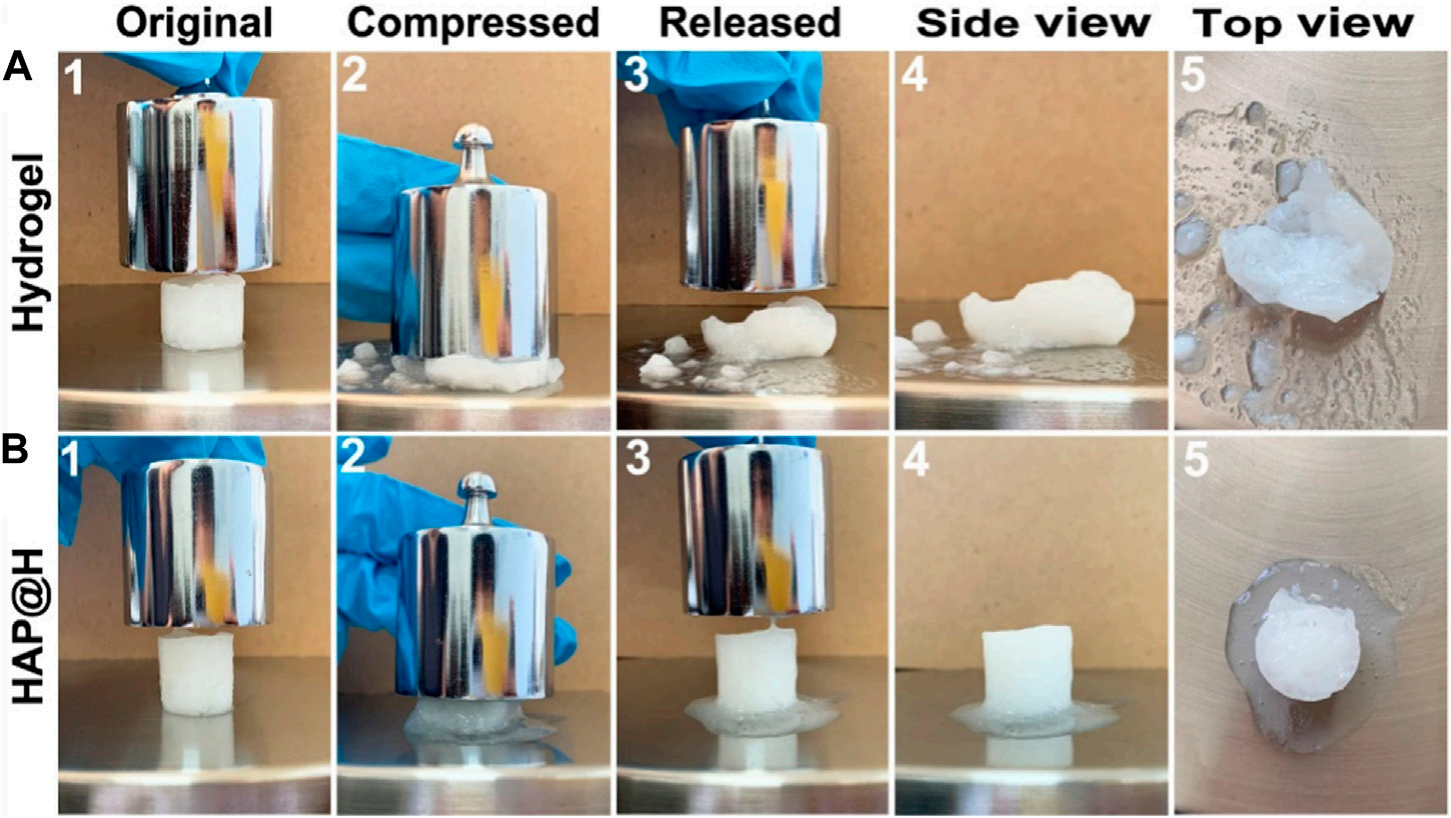
Compression test of pure hydrogel and HAP@H by a 200 g weight: **(A)** for pure hydrogel and **(B)** for HAP@H.

To investigate the compression properties of the obtained hydrogel, the obtained pure hydrogel and HAP@H were tested by a 200 g weight, as presented in Figure 10. It can be observed that the hydrogel sample had an original size of 16 mm in diameter and 18 mm in height [shown in Figure 10A(1)]. However, after the weight having compressed on the sample, Figure 10A(2) indicates that the gel block was crushed. After removing the weight, Figure 10A3) shows the gel sample’s structure had already been destroyed. The detailed information has been presented in Figure 10A(4) and Figure 10A(5). Unlike the structure of pure hydrogel block, which was crushed, after being compressed, the HAP@H gel block (indicated in Figure 10B) remained a complete structure. Except for the water in the gel being extruded, the structure of the gel remained intact.

**FIGURE 11:**
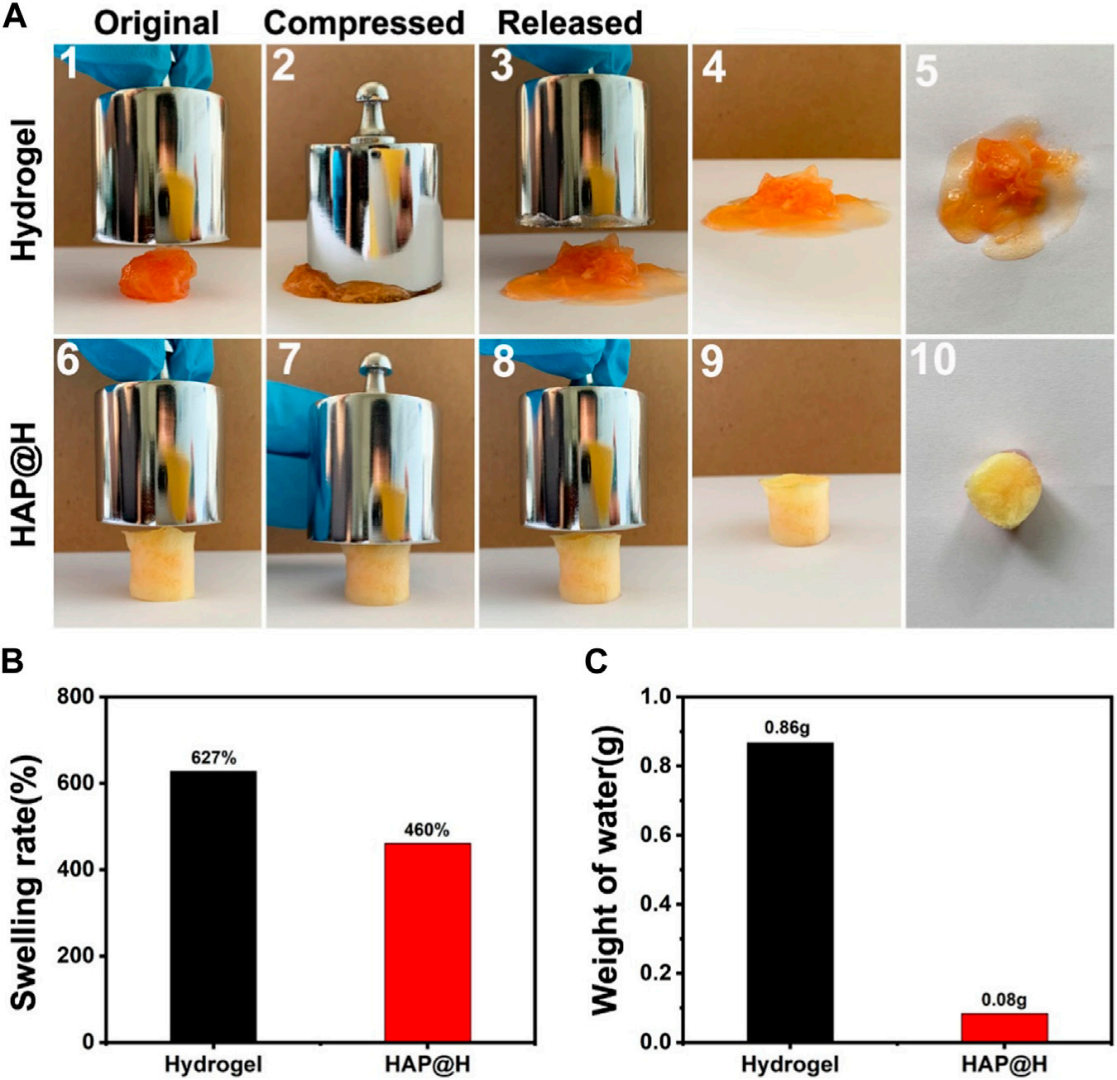
**(A)** Compression test of the lyophilized pure hydrogel and HAP@H by a 200 g weight: 1–5 for pure hydrogel and 6–10 for HAP@H; **(B)** swelling rate of the lyophilized pure hydrogel and HAP@H; **(C)** weight of water loss during compression test shown in (A) for pure hydrogel and HAP@H. In order to facilitate observation, Congo red was added into the water in swelling test for staining.

Figure 11 provides the swelling properties of the freezing dried hydrogel block. It is clear that the pure hydrogel swelled a lot, to a rate of 627%, by adsorbing plenty of water (Figure 11A (1–5) and Figure 11B). Especially, it was difficult for the swollen gel block to maintain a complete shape. After being squeezed (200 g weight), most of the water would be discharged (Figure 11C). Also, at the same time, the structure of pure hydrogel was broken. As for the lyophilized HAP@H, it had an appropriate swelling rate (Figure 11B). Furthermore, the photos in Figure 11A (6–10) clearly indicate that the HAP@H gel block maintained an intact shape under pressure of 200 g weight, and only little water had been squeezed out (Figure 11A(10) and Figure 11C). Especially, compared with Figure 10B2), Figure 11A7) clearly proves that the lyophilized HAP@H presented stronger compressive mechanical properties than freshly prepared HAP@H.

## 4 Conclusion

In summary, we have proposed a green approach of using ILs as a template to prepare HAP nanofibers. High-aspect ratio HAP nanofibers with 50 μm length were obtained. The morphology, size, and growth direction of HAP could be modulated by altering the alkyl chain length of ILs. The DSC results proved that HAP@H hydrogel had an increase (from 98°C to 107°C) in the water loss temperature compared to pure hydrogel. The compositing of HAP nanofibers into hydrogel significantly enhanced hydrogel performance. The testing and/or observation results suggest that HAP@H hydrogel presents a better porous structure, tensile performance, and compressive performance than that of pure hydrogel.

## Data Availability

The original contributions presented in the study are included in the article/Supplementary Material. Further inquiries can be directed to the corresponding author.
